# An autoantibody signature predictive for multiple sclerosis: evidence at the protein level and association with histopathological lesion types

**DOI:** 10.1007/s00415-026-13661-z

**Published:** 2026-04-08

**Authors:** S. Jarius, K. Ruprecht, I. Metz, F. B. König, J. Haas, W. Brück, F. Paul, B. Wildemann

**Affiliations:** 1 Division of Neuroimmunology, Department of Neurology, Otto Meyerhof Center, University of Heidelberg, Im Neuenheimer Feld 350, 69120 Heidelberg, Germany; 2 Department of Neuropathology, University of Göttingen, Göttingen, Germany; 3 Experimental and Clinical Research Center, a collaboration between the Max Delbrück Center for Molecular Medicine and Charité – Universitätsmedizin Berlin, Berlin, Germany; 4 NeuroCure Clinical Research Center, Charité – Universitätsmedizin Berlin, corporate member of Freie Universität Berlin, and Humboldt-Universität zu Berlin, and Berlin Institute of Health, Berlin, Germany

**Keywords:** Multiple sclerosis, MS, Epstein–Barr virus, EBV, Pathogenesis, Immunopathogenesis, Immunology, Histopathology, Autoantibodies, Antibodies, Immunoglobulin G, IgG, Pattern I MS, Pattern II MS, Pattern III MS, Microarray, Serum, Cerebrospinal fluid, CSF

## Abstract

Recently, an autoantibody signature considered to be predictive of multiple sclerosis (MS) has been reported in an article by Zamecnik et al. published in Nature Medicine, which is characterized by immunoglobulin G (IgG) responses to peptides sharing the amino acid motif P-(SA)-x-(SGA)-R-(SN)-(LRKH). These results are highly important, all the more so as the same motif is present also in two proteins expressed by Epstein–Barr virus (EBV), a pathogen that likely plays a key role in MS pathogenesis. However, clinically relevant autoantibody responses often target conformational epitopes, and peptides often differ from their corresponding proteins in terms of conformation. We were therefore interested in whether these findings can be reproduced at protein level and may thus play a role also in vivo. Here, we report findings from complementary experiments employing a microarray covering nearly 10,000 human full-length proteins and using serum and cerebrospinal fluid samples from patients with a histopathologically confirmed diagnosis of MS. Our data show that prominent IgG responses to full-length proteins bearing the P-(SA)-x-(SGA)-R-(SN)-(LRKH) motif can indeed be found in a substantial proportion of MS patients, although considerable inter-patient variability exists in both the type and number of individual responses. Notably, these IgG responses were more pronounced in patients with histopathologically defined pattern II MS (which is characterized by intralesional IgG and complement deposition) and pattern III MS than in patients with pattern I MS in our study. New motif-bearing candidate antigens identified in this study include RBMY2FP, CHMP2B, SRSF8 (SFRS2B), NUS1 (NgBR, Nogo-B receptor), and RTN2. Further studies investigating the diagnostic, pathophysiological, therapeutic, and prognostic implications of this antibody signature, as well as the potential role of cross-reactivity with EBV—suggested by the presence of the motif of interest in both EBV BRRF2 and EBV envelope glycoprotein M—are warranted and may significantly advance our understanding of MS.

**Supplementary Information:**

The online version contains supplementary material available at 10.1007/s00415-026-13661-z.

Dear Sirs,

Recently, a novel autoantibody signature in patients with multiple sclerosis (MS), characterized by immunoglobulin G (IgG) responses to peptides sharing the amino acid sequence P-(SA)-x-(SGA)-R-(SN)-(LRKH), has been proposed in a paper published in *Nature Medicine* [12]. These findings are highly interesting, all the more so as the motif in question is present also in two proteins of Epstein–Barr virus (EBV), a pathogen that plays a key role in MS pathogenesis as shown by others and us [1, 8–11].

However, clinically relevant autoantibody responses often target conformational epitopes [2]. It is therefore a notable drawback that (owing to the experimental setup chosen) only peptide fragments were used to probe the patients’ IgG repertoire in that study. Peptides often substantially differ in conformation from their corresponding full-length human proteins. Peptide-based serological assays are therefore known to be potentially problematic, in that they may lead to (false-positive or false-negative) results not reproducible in protein-based assays, and—together with methodologically suboptimal assay formats such as western blot or ELISA—have previously confounded research in autoantibodies, including in MS.

Believing that the MS community is interested in learning whether these—currently widely discussed—results can be reproduced at protein level (and may thus be relevant also in vivo), we here report our findings from complementary microarray experiments covering almost 10,000 human full-length proteins. Our data show that significant IgG responses to proteins bearing the proposed P-(SA)-x-(SGA)-R-(SN)-(LRKH) motif can indeed be found in a substantial proportion of MS patients although considerable variability exists between patients in the number and type of candidate proteins recognized. Notably, the IgG responses were more pronounced in patients with histopathologically defined [7] pattern II MS (which has previously been associated with an antibody-related pathogenesis) and pattern III MS than in pattern I MS in our study.

Several years ago, we investigated a cohort of patients with biopsy-supported MS (pattern II in 8; pattern III in 6; pattern I in 5; all negative for antibodies to myelin oligodendrocyte glycoprotein [MOG] and aquaporin-4 [AQP4] [4]; Supplementary File) for novel serum and cerebrospinal fluid (CSF) autoantibodies by means of a well-established commercial microarray employing human full-length proteins purified from a baculovirus-based expression system (ProtoArray 5.0, Invitrogen, Carlsbad, California, USA). This array has been successfully used by us and others in numerous studies to identify novel autoantigens in neurological and other diseases (e.g. [3, 5]). In response to the ongoing discussion of the paper by Zamecnik et al*.* in *Nature Medicine* [12], we conducted an in silico re-analysis of that cohort. For this purpose, all recorded antibody responses of each individual patient were ranked according to their Z factor [13], defined as Z = 1 – (3*σ*_*s*_ + 3*σ*_*c−*_)/(| *μ*_*s*_ – *μ*_*c−*_ |), after careful elimination of target antigens considered non-specific (see the Supplementary File for details).

In total, 14 proteins bearing the P-(SA)-x-(SGA)-R-(SN)-(LRKH) motif were present on the microarray (6 proteins corresponding to peptides already identified by Zamecnik et al*.* and 8 further proteins newly identified by us through screening of the primary sequences of all proteins on the microarray for that motif; see Supplementary Table 1) and 19 serum and 2 CSF samples from 19 adult patients with MS were probed, corresponding to 294 individual tests in total. The results were compared to those in 9 anonymized samples (8 × serum, 1 × CSF) from 8 adult patients with other inflammatory neurological diseases of the CNS, corresponding to another 126 tests.

Applying a strict Z factor cut-off of 0.8, 9% of the serum tests in the MS group were ‘hits’ (7 × RBMY2FP; 2 × CHERP; 4 × SRSF1; 3 × SRSF8; 1 × TRA2B [SRSF10]; 4 × CHMP2B, 2 × RTN2, 1 × NUS1), with some differences in frequency between the histopathological MS subgroups (12 ‘hits’ in 5/8 patients in the pattern II subgroup, 7 in 5/6 the pattern III subgroup, and 5 in only 1/5 in the pattern I subgroup), but none in the control group. Comparing the 6 proteins corresponding to peptides included in the original panel by Zamecnik et al*.* and the 8 newly identified additional motif-bearing proteins included in the microarray, an even higher hit rate was observed among the latter (11.2% vs. 4.35% of all tests at a 0.8 cut-off), further corroborating the significance of that motif.

Two patients (1 × pattern II, 1 × pattern I) showed a significant response to 5 motif-bearing proteins, 5 patients (3 × pattern II, 2 × pattern III) to 2, and 4 patients to 1 (1 × pattern II, 3 × pattern III). The median number of ‘hits’ per MS patient was 1 (range 0–5), with no hit in 8 patients (4 × pattern I, 3 × pattern II, 1 × pattern III). Thus, 11/19 (58%) patients responded to at least one of the 14 motif-bearing proteins and 7 (37%) to more than one. The two patients with the broadest range of significant serum IgG responses (to 5/14 proteins) recognized CHERP, SRSF1, SRSF8 (SRSF2B), CHMP2B, and RTN2; and CHERP, SRSF1, TRA2B (SFRS10), Nogo-B receptor (NUS1), and RBMY2FP, respectively. However, in summary, all patients recognized only a subset of proteins and some none, with the lack of reactivity in all but one pattern I MS patient being particularly noteworthy.

An application of individual Z factor cut-off values for each protein (ranging from the strict cut-off of 0.8 for some to a more conventional cut-off of 0.4 as recommended by the manufacturer) instead of a fixed cut-off for all proteins resulted in a hit rate of as much as 23% in the MS group vs. 0% in the control group, with one or more responses also in 4/5 patients with pattern I MS. In this scenario, 11 patients were positive for RTN2-IgG, 9 for SRSF8 (SFRS2B)-IgG, 5 for TRA2B (SFRS10)-IgG, 4 for SRSF1-IgG, 3 for SRFS7-IgG, 7 for RBMY2FP-IgG, 7 for ZRANB2-IgG, 7 for CHMP2B-IgG, 4 for EXO1-IgG, 3 for Nogo-B receptor (NUS1)-IgG and 2 for CHERP-IgG. Even at the lowest tested Z factor cut-off of 0.4, no patient showed an IgG response to MINK1, CD300LF or FAM134C. Notably, in all of the latter three proteins, and only in these, the P-(SA)-x-(SGA)-R-(SN)-(LRKH) motif is located outside of all potential B cell epitopes predicted by a BepiPred-2.0 analysis[6] (Supplementary Fig. 1). If these three proteins are excluded from the analysis, the rate of positive serum tests in the MS group increases to 30%. This compares to a frequency of 20–50% (median ~ 30%) of positive tests per peptide in the study by Zamecnik et al*.*

In the MS CSF samples tested, a high-rank CSF IgG response to the motif-bearing protein RBMY2FP (ranks 16 [pattern II MS] and 28 [pattern III MS], respectively) was noted, which was accompanied by a relatively highly ranked serum reactivity in one patient (rank 241). Notably, both samples were obtained during acute relapse. High-rank serum responses to RBMY2FP were also present in six additional MS patients (pattern II MS: ranks 53, 273, 279; pattern III MS: 136, 251, pattern I MS: 332; formally classified as ‘hit’ based on a conservative Z factor cut-off of 0.8 in all cases). In the pattern II MS patient, who showed the highest-ranking CSF response to RBMY2FP (rank 16), the corresponding serum IgG response rank was inversely low (rank 7934), raising the possibility of intrathecal synthesis of anti-RBMY2FP-IgG in this case.

Interestingly, the P-(SA)-x-(SGA)-R-(SN)-(LRKH) motif in TRA2B, a protein that yielded a relatively highly ranked serum IgG response (rank 359 of 9282) in one patient (pattern I; untreated) and was a formal ‘hit’ at a Z factor cut-off of 0.8, and in 4 further MS patients (but no control) at the manufacturer-recommended cut-off of 0.4, shares particularly high local sequence identity with the EBV protein BRRF2 (identical at 6/7 positions; PARSRSK vs. PAASRSK). The IgG response to the corresponding peptide 456,870 was among the most prevalent responses in the study by Zamecnik et al*.*

Notably, several motif-bearing target antigens identified in the microarray are also expressed by neuronal and/or glial cells, and some of these antigens have been implicated in neurological disease (see Supplementary File for details). It is also noteworthy that variants of the motif P-(SA)-x-(SGA)-R-(SN)-(LRKH) and even the exact motif sequence contained in the EBV protein BRRF2 is present in UniProtKB/TrEMBL entries from several microbial species beyond EBV (including *Pseudomonas *spp. and *Aspergillus *spp.), which is of interest in light of the frequent occurrence of MS relapses in postinfectious contexts, and commonly consumed plant species, including soya bean and maize; that some motif-matching sequences (including the not previously described sequence PSRSRSR) occur in more than one protein; and, finally, that STRING analysis showed significant protein-protein interaction enrichment among several of the motif-bearing proteins (see Supplementary File for details).

It is an obvious limitation that not all proteins corresponding to peptides identified by Zamecnik et al. were present on our microarray; accordingly, our data may even underestimate the true extent of reactivity to and cross-reactivity among proteins bearing the motif of interest. Sample size is another limitation, and confirmation in larger, independent cohorts is required. Among the particular strengths of this study are the high number of proteins tested and the fact that the diagnosis of MS was histologically confirmed in our patients. An extended, 15-page version of this letter with description of methods, further results, tables and figures, and a more detailed discussion of the individual antibody reactivities/target antigens identified in this study can be found in the Supplementary File.

In conclusion, the high frequency of IgG antibody responses against at least one of the proteins, as observed in our microarray experiments, provides evidence in favor of the motif identified by Zamecnik et al*.* in a peptide-based approach being of relevance also at the level of full-length proteins. Further studies investigating the diagnostic, pathophysiological, therapeutic, and prognostic implications of this antibody signature as well as the role of cross-reactivity with EBV, strongly suggested by the presence of the motif of interest both in EBV-BRRF2 and EBV envelope glycoprotein M, are warranted and may significantly advance our understanding of MS.

## Supplementary Information

Below is the link to the electronic supplementary material.

Supplementary file1 (PDF 2017 KB)

## Data Availability

All microarray data on P-(SA)-x-(SGA)-R-(SN)-(LRKH) motif-bearing proteins analyzed and discussed in this paper are available from the authors upon reasonable request by any interested researcher. This includes a full list of proteins on the microarray, accession numbers, sequences used, and all individual Z factor and rank results. The microarray experiments also led to the identification of novel antigenic protein targets in histopathologically defined subsets of MS patients. These targets are neither the subject of this article nor of the study by Zamecnik et al., and they do not bear the P-(SA)-x-(SGA)-R-(SN)-(LRKH) motif (unpublished data; publication in preparation). Therefore, the authors kindly ask for readers’ understanding that access to individual Z factor and rank results for proteins unrelated to this article or that by Zamecnik et al., but co-present on the microarray, cannot currently be provided for confidentiality reasons.

## Abbreviations

AQP4: Aquaporin-4
EBV: Epstein–Barr virus
ELISA: Enzyme-linked immunosorbent assay
IgG: Immunoglobulin G
MOG: Myelin oligodendrocyte glycoprotein
MS: Multiple sclerosis
