# Supplementary material for: An autoantibody signature predictive for multiple sclerosis: evidence at the protein level and association with histopathological lesion types
Source: J Neurol. 2026 Apr 8;273(5):257. doi: 10.1007/s00415-026-13661-z (PMC13061806; doi:10.1007/s00415-026-13661-z)
Supplement: Supplementary file 1 — Supplementary file1 (PDF 2017 KB) [file 415_2026_13661_MOESM1_ESM.pdf]

Journal of Neurology (2026)
DOI 10.1007/s00415-026-13661-z

ELECTRONIC SUPPLEMENTARY MATERIAL

An autoantibody signature predictive for multiple sclerosis: evidence at the protein level and association with histopathological lesion types

Jarius S[1], Ruprecht K[1], Metz I[2], König FB[2], Haas J[1], Brück W[2], Paul F[3,4], Wildemann B[1].

Received: 15 January 2026 / Revised: 26 January 2026 / Accepted: 27 January 2026
© The Author(s) 2026

Abstract Recently, an autoantibody signature considered to be predictive of multiple sclerosis (MS) has been reported in an article published in *Nature Medicine*, which is characterized by immunoglobulin G (IgG) responses to peptides sharing the amino acid motif P-(SA)-x-(SGA)-R-(SN)-(LRKH). These results are highly important, all the more so as the same motif is present also in two proteins expressed by Epstein-Barr virus (EBV), a pathogen that likely plays a key role in MS pathogenesis. However, clinically relevant autoantibody responses often target conformational epitopes, and peptides often differ from their corresponding proteins in terms of conformation. We were therefore interested in whether these findings can be reproduced at protein level – and may thus play a role also *in vivo*. Here, we report findings from complementary experiments employing a microarray covering nearly 10,000 human full-length proteins and using serum and cerebrospinal fluid samples from patients with a histopathologically confirmed diagnosis of MS. Our data show that prominent IgG responses to full-length proteins bearing the P-(SA)-x-(SGA)-R-(SN)-(LRKH) motif can indeed be found in a substantial proportion of MS patients, although considerable inter-patient variability exists in both the type and number of individual responses. Notably, these IgG responses were more pronounced in patients with histopathologically defined pattern II MS (which is characterized, among other features, by IgG and complement deposition) and pattern III MS than in patients with pattern I MS in our study. New motif-bearing candidate antigens identified in this study include RBMY2FP, CHMP2B, SRSF8 (SFRS2B), NUS1 (NgBR, Nogo-B receptor), and RTN2. Further studies investigating the diagnostic, pathophysiological, therapeutic, and prognostic implications of this antibody signature, as well as the role of cross-reactivity with EBV—strongly suggested by the presence of the motif of interest in both EBV BRRF2 and the EBV envelope glycoprotein M—are warranted and may significantly advance our understanding of MS.

✉ S. Jarius and B. Wildemann
sven.jarius@med.uni-heidelberg.de; brigitte.wildemann@med.uni-heidelberg.de
Division of Neuroimmunology, Department of Neurology, University of Heidelberg, Otto Meyerhof Center, Im Neuenheimer Feld 350, D-69120 Heidelberg, Germany

Keywords Multiple sclerosis · MS · Epstein-Barr virus · EBV · Pathogenesis · Immunopathogenesis · Immunology · Histopathology · Autoantibodies · Antibodies · Immunoglobulin G · IgG · Pattern I MS · Pattern II MS · Pattern III MS · Microarray · Serum · Cerebrospinal fluid · CSF

Abbreviations

AQP4	Aquaporin-4
DTT	Dithiothreitol
EBV	Epstein–Barr virus
ELISA	Enzyme-linked immunosorbent assay
HEPES	4-(2-hydroxyethyl)-1-piperazineethanesulfonic acid
IgG	Immunoglobulin G
IQR	Interquartile range
MOG	Myelin oligodendrocyte glycoprotein
MS	Multiple sclerosis
OIND	Other inflammatory neurological diseases
PBS	Phosphate-buffered saline
PPI	Protein-protein interaction

Dear Sirs,

Recently, a novel autoantibody signature in patients with multiple sclerosis (MS) has been reported, characterised by immunoglobulin G (IgG) responses to peptides sharing the amino acid sequence P-(SA)-x-(SGA)-R-(SN)-(LRKH) [30]. These findings are highly interesting, all the more so as the motif is present also in two proteins of Epstein-Barr virus (EBV), a pathogen that plays a key role in the pathogenesis of MS as shown by others and us [1, 18, 20, 21, 23]. However, clinically relevant autoantibody responses often target conformational epitopes [5]. It is therefore a notable limitation that (owing to the experimental setup chosen) only peptide fragments were used to probe the patients' IgG repertoire in that study. Peptides often substantially differ in conformation from their corresponding full-length human proteins. Peptide-based serological assays are therefore known to be potentially problematic, in that they may lead to (false-positive or false-negative) results not reproducible in protein-based assays and – together with methodologically suboptimal assay formats such as western blot or enzyme-linked immunosorbent assays (ELISA) – have previously contributed to misleading results in MS autoantibody research. We were therefore interested in whether these – currently widely discussed – results can be reproduced at protein level – and may thus play a role also *in vivo*. Here, we report on our findings from complementary microarray experiments employing almost 10,000 human full-length proteins. Our data show that prominent IgG responses to full-length proteins bearing the P-(SA)-x-(SGA)-R-(SN)-(LRKH) motif can indeed be found in a subset of MS patients. Of note, these IgG responses were more pronounced in patients with histopathologically defined pattern II and pattern III MS than in pattern I MS [15].

Several years ago, we screened a cohort of patients with biopsy-supported MS [11] for serum and cerebrospinal fluid (CSF) IgG autoantibodies by means of a well-established commercial microarray of human proteins purified from a baculovirus-based expression system (ProtoArray 5.0, Invitrogen, Carlsbad, California, USA). This array has been successfully used by us and others in numerous studies to identify novel autoantigens in neurological and other diseases (e.g. [6, 12]). Notably, this cohort included also 8 patients with pattern II MS, a histopathological subtype characterised by intralesional IgG and complement deposition, suggesting a possible autoantibody-related pathogenesis, besides 5 patients with pattern I MS and 6 with pattern III MS. All patients were negative for IgG antibodies against myelin oligodendrocyte glycoprotein (MOG) and aquaporin-4 (AQP4). In response to the ongoing discussion of the paper by Zamecnik *et al.* in *Nature Medicine* we conducted an *in silico* re-analysis of that cohort.

In total, 14 proteins bearing the motif P-(SA)-x-(SGA)-R-(SN)-(LRKH) were included on the microarray (6 proteins corresponding to peptides identified in [30] and 8 additional proteins newly identified by us through screening of the sequences of all proteins on the microarray for the motif in question; Supplementary Table 1) and 21 samples (19×serum, 2×CSF) from 19 adult patients with MS according to the McDonald criteria (male:female ratio, 1:2.2; median age 44 years) were probed, amounting to 294 individual tests in total. The results were compared to those from 9 samples (8×serum, 1×CSF) obtained from 8 anonymised adult control patients with other inflammatory neurological diseases of the CNS (OIND) who did not meet the McDonald criteria for MS, corresponding to another 126 tests. All samples were provided by the Department of Neuropathology, University of Göttingen, Germany, with the exception of two MS serum and CSF samples and the control samples, which were collected at the Department of Neurology, University of Heidelberg, Germany. The study was approved by the institutional review boards of the medical faculties of the University of Göttingen and the University of Heidelberg, and patients gave written informed consent or were analysed in anonymised fashion as required by the institutional review board of the University of Heidelberg.

The commercially available human protein microarray, spotted with 9,482 human full-length proteins (all spotted in duplicate) purified from a baculovirus-based expression system, was probed with the patients' serum or cerebrospinal fluid (CSF) samples according to the manufacturer's instructions [12]. Briefly, the array was incubated for 1 h at 4°C with blocking buffer containing 50 mM HEPES, pH 7.5, 200 mM NaCl, 0.08% triton X-100, 25% glycerol, 20 mM reduced glutathione, 1 mM DTT, and a commercial synthetic block (Invitrogen).

After washing, the array was incubated with the patient's serum at a 1:1,500 dilution or CSF at a 1:10 dilution in washing buffer containing PBS-0.1% Tween and synthetic block. Following additional washing steps, a goat anti-human IgG detection antibody labelled with AF647 (final concentration 1 µg/ml; Invitrogen, catalogue number A-21445) was applied for 90 min at 4°C. A Genepix™ 4000 B microarray scanner and corresponding application software (Genepix, Sunnyvale, CA) was used to scan the slide. ProtoArray v5.0 Prospector software (Invitrogen) was employed to calculate fluorescent signal values, including corrections for background and negative controls present on the microarray, and to calculate Z factors, defined as $Z = 1 - (3\sigma_s + 3\sigma_{c-})/(|\mu_s - \mu_{c-}|)$, where σ_s = signal sample standard deviation for the

protein features; σ_{c-} = signal sample standard deviation for the negative control features; μ_s = mean signal for the protein features; μ_{c-} = mean signal for the negative control features [31]. A 'hit' was defined as any protein for which the Z factor exceeded a pre-defined cut-off. All recorded antibody responses from a given patient were then ranked according to their Z factor. Proteins listed by the manufacturer as causing non-specific responses (i.e., regularly present in healthy individuals and across a broad range of diseases), many of which belong to the immunoglobulin superfamily, as well as proteins causing 'hits' in all MS patients *and* in at least 7/8 control patients in the present cohort were excluded from the ranking for the purpose of this study.

Applying a strict Z factor cut-off of 0.8, 9% of the 266 serum tests in the MS group were 'hits' (7×RBMY2FP [RNA binding motif protein Y-linked family 2 member F]; 2×CHERP [calcium homeostasis endoplasmic reticulum protein]; 4×SRSF1 [splicing factor, arginine/serine-rich 1, transcript variant 1]; 3×SRSF8 [splicing factor, arginine/serine-rich 8], also termed SFRS2B; 1× TRA2B [transformer-2 protein homolog beta], also termed SFRS10; 4×CHMP2B [charged multivesicular body protein 2B], 2×RTN2 [reticulon 2], 1×NUS1 [Nogo-B receptor]), with some differences between the histopathological MS subgroups (12 'hits' in 5/8 patients in the pattern II subgroup; 7 in 5/6 the pattern III subgroup, and 5 in only 1/5 in the pattern I subgroup), but none in the control group (Supplementary Table 2). Comparing the 6 proteins corresponding to peptides included in the original panel by Zamecnik *et al.* and the 8 newly identified additional motif-bearing proteins included in the microarray, an even higher 'hit rate' was observed with the latter (11.2% vs. 4.35% of all tests at a 0.8 cut-off), further corroborating the significance of that motif.

Two patients (1×pattern II, 1×pattern I) showed significant responses to 5 motif-bearing proteins, 5 patients (3×pattern II, 2×pattern III) to 2 proteins, and 4 patients to 1 protein (1×pattern II, 3×pattern III). The median number of 'hits' per MS patient was 1 (range 0-5), with no hit in 8 patients (4×pattern I, 3×pattern II, 1×pattern III). Thus, 11/19 (58%) patients responded to at least one of the 14 proteins and 7 (37%) to more than one protein. The two patients with the broadest range of significant serum IgG responses (to 5/14 proteins), recognised CHERP, SRSF1, SRSF8 (SFRS2B), CHMP2B, and RTN2; and CHERP, SRSF1, TRA2B (SFRS10), Nogo-B receptor (NUS1), and RBMY2FP, respectively. However, in summary, all patients recognised only a subset of proteins and some none, with the lack of reactivity in all but one pattern I patient being particularly notable.

Application of individual Z factor cut-off values for each protein (ranging from the strict cut-off of 0.8 for some to a more conventional cut-off of 0.4 as recommended by the manufacturer) instead of a fixed cut-off for all proteins resulted in a 'hit rate' of as much as 23% in the MS group vs. 0% in the control group, with one or more responses also in 4/5 patients with pattern I MS. In this scenario, 11 patients were positive for RTN2-IgG, 9 for SRSF8 (SFRS2B)-IgG, 5 for TRA2B (SFRS10)-IgG, 4 for SRSF1-IgG, 3 for SRSF7-IgG, 7 for RBMY2FP-IgG, 7 for ZRANB2-IgG, 7 for CHMP2B-IgG, 4 for EXO1-IgG, 3 for Nogo-B receptor (NUS1)-IgG, and 2 for CHERP-IgG. Even at the lowest tested Z factor cut-off of 0.4, no patient showed an IgG response to MINK1, CD300LF, or FAM134C. If these three proteins were excluded from the analysis, the rate of positive serum tests in the MS group increased to 30%. It is obvious that the control group was not sufficiently large to allow determining cut-offs for the various proteins in a definite way; more studies are needed to better understand what protein panel and cut-offs to use for best prediction. The 30% detection rate in our cohort compares to a frequency of 20-50% (median ~30%) of positive tests per peptide in the study by Zamecnik *et al.* However, this comparison should be interpreted with caution, as the two studies differ in assay format, antigen presentation, and cut-off definition.

As in that previous study, considerable inter-patient variability in individual responses was observed. RBMY2FP was recognised by most patients when applying a strict cut-off of 0.8 and RTN2 when using individual cut-offs for each protein. RBMY2FP, which contains the P-(SA)-x-(SGA)-R-(SN)-(LRKH) motif sequence PASSRSR, elicited the highest-ranking serum IgG response in 8/19 (42%) patients (median rank 256 of 9282). Consequently, among the 14 proteins tested, RBMY2FP was the most frequently top-ranked protein at the 0.8 cut-off. In line with our findings, Zamecnik *et al.* previously identified a response to peptide 64485, corresponding to the closely related protein RBMY1B, in approximately 30%. RBMY1B bears a similar yet not identical sequence (PASSRNR); RBMY1B and other members of the RBMY1 family – all of which contain the motif in question – were not included in the microarray. Given that the corresponding gene is located on the Y chromosome (and MS is a disease with female predominance) and that RBMY2FP is a (possibly [14] non-translated) pseudogene, RBMY2FP is unlikely to represent the primary *in vivo* antigenic driver.

The only two patients for whom matched CSF samples were available both exhibited a high-rank CSF IgG response to RBMY2FP (rank 16 [pattern II MS] and rank 28 [pattern III MS], respectively, out of 9282 responses assessed). These were also the highest-ranking CSF responses to any of the 14 proteins. Notably, both samples were obtained during acute relapse (as opposed to most of the remaining samples; Supplementary Table 2). In the pattern III patient, this

CSF response was accompanied by a relatively high-ranking serum reactivity to RBMY2FP (rank 241). High-ranking serum IgG responses to RBMY2FP were also noted in six additional MS patients (pattern II MS: ranks 53, 273, 279; pattern III MS: 136, 251; pattern I MS: 332), all formally classified as 'hits' based on a conservative Z factor cut-off of 0.8. In contrast, no similarly high ranks were observed in the 8 control subjects in serum (median rank 2172).

Of note, in the pattern II MS patient with the highest-ranking CSF response to RBMY2FP (rank 16), the corresponding serum IgG response ranked inversely low (rank 7,934), raising the possibility of intrathecal synthesis of anti-RBMY2FP-IgG. Intrathecal IgG synthesis is a hallmark of MS, present in >90% of cases. Interestingly, amino acids 96-102 of RBMY2FP, corresponding to the PASSRSR motif sequence, yielded the highest scores in a BepiPred Linear Epitope Prediction 2.0 analysis [13], at a strict (sensitivity 0.00182, specificity 0.99954) threshold of 0.7 among all amino acids in that protein (Supplementary Figure 1). Emini analysis predicted surface accessibility of the motif [4]. No significant response to RBMY2FP – or any other of the motif-bearing proteins included in the microarray – were observed in the control CSF sample tested (from a patient with anti-septin 3-positive cerebellitis [17]), irrespective of whether a Z factor cut-off of 0.8 or 0.4 was applied.

Notably, several motif-bearing target antigens identified in the microarray experiments are also expressed by neuronal and/or glial cells, some of which have been implicated in neurological disease, and are therefore of potential pathophysiological interest:

(i) Two patients exhibited high-rank serum IgG responses (ranked 42 [pattern II] and 118 [pattern I] among 9282 responses) to CHERP. CHERP corresponds to peptide 554788 identified by Zamecnik *et al.* as an IgG target in approximately 20% of samples. The corresponding gene was prioritized as one of 37 candidate susceptibility loci/genes in a network-based multiple sclerosis pathway analysis of genome-wide association study data [8]. However, CHERP expression shows low tissue, brain regional and brain cell type specificity [28].

(ii) Reticulon-2, also termed neuroendocrine-specific protein-like 1, elicited a relatively strong response in two patients at a Z factor cut-off of 0.8 (ranks 302 and 358; both with pattern II MS; treated with interferon beta [IFN-β]). Spastic paraplegia 12 is associated with mutations in the RTN2 gene on chromosome 19q13. Moreover, RTN2 is a member of the reticulon family and is related to RTN4, which encodes Nogo, the isoform A of which is a neurite outgrowth inhibitor and oligodendrocyte membrane protein [2]. Autoantibodies to Nogo-A have been repeatedly discussed as playing a role in the pathogenesis of MS, particularly in pattern II MS [19, 25]. However, reticulon-2 and reticulon-4A (Nogo-A) share only limited sequence identity (31.6%), and the latter does not contain the P-(SA)-x-(SGA)-R-(SN)-(LRKH) motif. If cross-reactivity between reticulon-2 and Nogo-A existed, it would thus be mediated by antibodies to epitopes outside that motif.

(iii) NUS1 (also termed NgBR) functions as a Nogo-B receptor, which is structurally distinct from RTN4R/NgR1, a receptor for Nogo-A/Nogo-66. NUS1 has been identified as a candidate gene in Parkinson's disease [7] and pathogenic variants have been associated with neurodevelopmental disorders [26]. Moreover, Schwann cell-expressed Nogo-B has been reported to modulate axonal branching of adult sensory neurons through NUS1/NgBR [3].

(iv.) CHMP2B (3×pattern II MS, 1×pattern I, but highly ranked only in one patient [rank 103] with pattern II, who was treated with IFN-β) is expressed in neurons across major brain regions and shows prominent oligodendrocyte/white-matter-associated RNA expression in HPA brain datasets [24, 28].

(v.) Finally, the P-(SA)-x-(SGA)-R-(SN)-(LRKH) motif in TRA2B (SFRS10), a protein that yielded a relatively highly ranked response (rank 359 of 9282) in one patient (pattern I; untreated) and was a formal 'hit' at a Z factor cut-off of 0.8 and in 4 further MS patients at the manufacturer-recommended cut-off of 0.4 (with no 'hits' in the control group), shares particularly high local sequence identity with the EBV protein BRRF2 (identical at 6/7 positions; PARSRSK vs. PAASRSK). Among all proteins identified either in our assay or in the peptide-based assay as being recognised by MS patient IgG, only DENND4C (DENN domain-containing protein 4C; not included in the microarray) shows a similar degree of linear motif sequence homology with EBV-BRRF2 (PAVSRSK; note that the Gonnet PAM 250 matrix assigns a higher score to the substitution of alanine with valine than to the substitution of alanine with arginine, indicating even higher similarity of DENND4C than of TRA2B to BRRF2). The IgG response to the corresponding peptide 456,817 was also one of the five most prevalent responses in the study by Zamecnik *et al.* Infection with EBV is considered a *conditio sine qua non* for the development of MS, and cross-reactivity between EBV and human proteins has been proposed as a possible mechanism involved in MS pathogenesis [1, 18, 20, 21, 23]. A BepiPred-2.0 analysis showed that the TRA2B-specific motif sequence lies within a predicted B cell epitope [13] (Supplementary Figure 1). In addition, Emini surface accessibility prediction [4] suggested that this region is likely surface-exposed.

Interestingly, in all three proteins (and only in these) that did not meet the response threshold in any of the MS patients

or controls (MINK1, CD300LF, FAM134C), even at a Z factor cut-off of 0.4, the P-(SA)-x-(SGA)-R-(SN)-(LRKH) motif is located outside of all B cell epitopes predicted by BepiPred-2.0 [13] (Supplementary Figure 1). Notably, the highest median BepiPred-2.0 prediction score of all amino acids located in the motif among the 14 proteins analysed was found for RBMY2FP (0.741) and the lowest for MINK1 (0.567), CD300LF (0.579) and FAM134C (0.517) (not shown).

Of further note, STRING analysis of the submitted protein set showed protein-protein interaction (PPI) enrichment among several of the motif-bearing proteins, with a PPI enrichment p-value of 1.42×10^{-5} (see Supplementary Figure 2) [27, 29]: Specifically, RBMY-family proteins, including RBMY1B and closely related paralogs, and SRSF8 (also known as SFRS2B) both interact with TRA2B (also referred to as SFRS10) and with SRSF3. TRA2B in turn binds to the A3 enhancer protein SRSF4 and interacts also with SRSF1 [29]. Additionally, ZRANB2 is a splice factor required for alternative splicing of TRA2B/SFRS10 transcripts [29], and RBMY1A1 and RBMY1C have been shown to act additively with TRA2B to promote exon 7 inclusion of SMN (survival of motor neuron) [29].

A substantial proportion of MS relapses occur in post-infectious contexts [16]. Zamecnik *et al.* already pointed out that variants of the motif P-(SA)-x-(SGA)-R-(SN)-(LRKH) are also present in a range of human pathogens beyond EBV. This raises the possibility that infection with such microbes may trigger immune reactivation in MS. We would like to add that even the exact EBV BRRF2 heptapeptide (i.e., PAASRSK) is present in UniProtKB/TrEMBL entries from several microbial species, with or without pathogenic relevance in humans, including *Pseudomonas* spp., *Aspergillus* spp., and *Penicillium* spp.

The exact EBV BRRF2 motif sequence might also be present in a few commonly consumed plant species [29]. Specifically, the motif occurs in unreviewed UniProtKB/TrEMBL entries annotated as non-specific serine/threonine protein kinases in *Glycine max* (K7KAS3) and *Glycine soja* (A0A0B2SWB6) – cultivated and wild soybean, respectively – as well as in a DnaJ domain-containing maize protein (B6SUG3; evidence at transcript level) [29]. The relevance of this finding is currently unknown but may warrant further investigation. In theory, dietary exposure to such motif-bearing plant proteins in individuals with IgG responses to the motif could have varying effects, including tolerance induction, immune reactivation, or no immunological consequences at all.

The most frequently observed immune response (at the conservative cut-off of 0.8) in our microarray experiments was directed against RBMY2FP. Members of the RBMY (RNA-binding motif Y) gene family are believed to function as splicing regulators during spermatogenesis and are required for normal sperm development. Multiple closely related RBMY1A paralogs – sharing substantial sequence identity with RBMY2FP (see Supplementary Figure 3) – are located in a gene cluster within the AZFb azoospermia factor region of the Y chromosome. Given previous reports of impaired spermatogenesis in males with MS – characterized by reduced sperm count and motility, as well as altered sperm morphology [22] – and the high prevalence of antibodies to PASSR-(SN)-R-bearing substrates observed in our study at the protein level (RBMY2FP) and by Zamecnik *et al.* at the peptide level (RBMY1B), further investigations into a potential link between autoimmunity (including T cell-mediated autoimmunity) to RBMY proteins and spermatogenetic dysfunction in MS are of potential interest.

None of the patients or controls showed a significant IgG response to heterogeneous nuclear ribonucleoprotein G (hnRNP G) at a cut-off of 0.4. HnRNP G, encoded by RBMX, the functional X-linked homolog (gametolog) of the RBMY gene family, does not contain the P-(SA)-x-(SGA)-R-(SN)-(LRKH) motif.

For completeness, we would like to point out that SRSF4, from which the top-ranked motif-containing peptides in Fig. 3a of Zamecnik *et al.* were derived, contains even two distinct sequence segments matching the P-(SA)-x-(SGA)-R-(SN)-(LRKH) motif: PSESRSR at position 455 of the canonical sequence, as already mentioned by Zamecnik *et al.*, and PSRSRSR at position 483, not previously discussed. Of note, the latter sequence (PSRSRSR) is present also in CHERP, PPIG and SRSF7. In our search of the reviewed human UniProt Knowledgebase (UniProtKB)/Swiss-Prot proteome, we did not identify this exact heptapeptide in any other human protein.

When analysing the total MS cohort rather than individual responses, it was notable that median IgG responses were generally low at the group level. Across all 14 candidate proteins, the overall median rank of all responses was just 3330, and median Z factor ranks for the individual proteins (based on data from 19 MS patients) ranged from 1037 (for SRSF1) to 9155 (for MINK1, misshapen-like kinase 1, transcript variant 4), with an IQR of 1322-5069. This distribution did not differ markedly from that observed in the 8 control samples, where the overall median Z factor rank was 3559, and individual ranks ranged from 513 (SRSF1) to 7704 (MINK1) (IQR 2246-5014). No significant differences were observed between the three MS subgroups when considering all 14 proteins (median of all Z factor ranks: pattern II MS 3451 vs. pattern III 3273 vs. pattern I 3356). In the two available CSF samples (one from a patient with pattern

II and one with pattern III MS), IgG responses to the candidate proteins were also weak, with overall median ranks of 3626 and 2945, respectively.

However, we did not expect all patients to recognise all proteins and all to the same extent. Several factors help explain this:

(a) Cross-reactivity was already limited at peptide level in the study by Zamecnik *et al.*, and even less cross-reactivity was anticipated at the protein level due to potential differences in conformation between peptides and full-length proteins.

(b) Exact epitope specificities (and therefore the potential for cross-reactivity) as well as titres and affinities are likely to vary between individual patients.

(c) Given prior evidence of heterogeneity in MS, it is conceivable that immune recognition with the motif of interest plays a role only in specific MS subgroups.

(d) Although protein-based assays preserve more conformational integrity than peptide-based assays, they still do not fully replicate *in vivo* conditions. Factors such as purification effects, differences in posttranslational modification, and lack of physiological protein–protein or protein–lipid interactions – as well as the artificial conditions of the microarray platform – can all affect antibody binding.

In addition, differences in disease duration may influence the extent of immune reactivity. In chronic autoimmunity, affinity maturation and epitope spreading can lead to stronger and broader responses, potentially increasing cross-reactivity.

Of note in this context, the full-length SH3 domain-binding protein 2 (SH3BP2) – which corresponds to peptide target 216602 identified by Zamecnik *et al.* in nearly 40% of their samples – ranked among the top 50 serum IgG responses (out of 9482) in two samples (ranks 22 and 35), despite the fact that the isoform used on the microarray (isoform a, transcript variants 1 and 2) did not include the P-(SA)-x-(SGA)-R-(SN)-(LRKH) motif (Supplementary Figure 4). Given the high rate of IgG binding to a SH3BP2 peptide bearing the motif in the study by Zamecnik *et al.*, this finding suggests that epitope spreading to regions outside the original motif may occur in some patients.

The present study has both strengths and limitations. A key strength is that the MS cohort consisted exclusively of patients with a histopathologically supported diagnosis (moreover, major differential diagnoses – neuromyelitis optica spectrum disorder [11] and myelin oligodendrocyte glycoprotein antibody-associated disease [10] – were serologically excluded in all patients, and a very broad spectrum of other antibody-associated types of autoimmune encephalitis was ruled out in most cases [9]). However, this might also have introduced bias, as biopsies are typically performed in patients with a particularly severe disease course. Additional limitations include, besides the sample size, the fact that most samples were obtained during clinical remission, and CSF was available from only two patients. We acknowledge that while the use of full-length proteins increases the likelihood of preserving native protein conformation, it does not guarantee it in all cases. Finally, it is an obvious limitation that not all proteins corresponding to the peptides identified by Zamecnik *et al.* were present in the microarray. As a result, our data may even underestimate the extent of reactivity to – and cross-reactivity among – proteins bearing the motif of interest.

In conclusion, the high frequency of IgG antibody responses against at least one of the motif-bearing proteins in patients with MS, as observed in our study, provides evidence in favour of the motif identified by Zamecnik *et al.* in a peptide-based approach being of relevance also at the level of full-length proteins, particularly in pattern II and pattern III MS. Further studies investigating the diagnostic, pathophysiological, therapeutic and prognostic implications of this antibody signature are warranted. In addition, research into potential reactivity (or cross-reactivity) with further motif-bearing full-length proteins whose relevant epitopes may not be captured by peptide-based assays because they depend on native protein conformation, as well as studies exploring the potential link to EBV – suggested by the presence of the motif of interest both in EBV BRRF2 and EBV envelope glycoprotein M (gM) – may provide important novel insights into MS pathogenesis.

Acknowledgment The authors are grateful to Mrs. Anna Eschlbeck and Mrs. Brigitte Fritz for excellent technical assistance.

Author contributions S.J. and B.W. conceived the study. S.J. analysed the microarray data, conducted the bioinformatic analyses (including identification of additional candidate proteins, B-cell linear epitope prediction and surface expression prediction), conducted the serological tests, and wrote the manuscript. W.B., I.M. and F.B.K. performed the histopathological studies. B.W. and K.R. were involved in patient care, and B.W., K.R. and J.H. in supervising the project. All authors were involved in revising the manuscript for intellectual content.

Funding There was no specific funding for this study.

Data availability All microarray data on P-(SA)-x-(SGA)-R-(SN)-(LRKH) motif-bearing proteins analysed and discussed in this paper are available from the authors upon reasonable request by any interested researcher. This includes a full list of proteins on the microarray, accession numbers, sequences used, and all individual Z factor and rank results. The microarray experiments also led to the identification of novel antigenic protein targets in histopathologically defined subsets of MS patients. These targets are neither the subject of this article nor of the study by Zamecnik *et al.*, and they do not bear the P-(SA)-x-(SGA)-R-(SN)-(LRKH) motif (unpublished data; publication in preparation). Therefore, the authors kindly ask for readers' understanding that access to individual Z factor and rank results for proteins unrelated to this article or that by Zamecnik *et al.*, but co-present on the microarray, cannot currently be provided for confidentiality reasons.

Declarations

Conflicts of interest S.J. reports no competing interests. K.R. received research support from German Ministry of Education and Research, European Union (821283-2), Stiftung Charité, Novartis, Merck Serono, Guthy-Jackson Charitable Foundation, and Arthur Arnstein Foundation and speaker's honoraria from Virion Serion and Novartis; none related to this study. I.M. reports personal fees from Sanofi, BiogenIdec, Bayer Healthcare, TEVA, Serono, Novartis, Genzyme, Roche, and grants from BiogenIdec, Genzyme, Sanofi; none related to this study. F.B.K. reports no competing interests. J.H. reports no competing interests. W.B. reports no competing interests. F.P. reports research support from Bayer, Novartis, Biogen, Teva, Sanofi-Aventis/Genzyme, Alexion, and Merck Serono and research support from the German Research Council, Werth Stiftung of the City of Cologne, German Ministry of Education and Research, Arthur Arnstein Stiftung Berlin, EU FP7 Framework Program, Guthy-Jackson Charitable Foundation, and NMSS; he also reports consulting fees as an associate editor for Neurology, Neuroimmunology & Neuroinflammation and as an academic editor for PloS ONE and consultant fees for Sanofi Genzyme, Biogen, MedImmune, Shire, and Alexion; he also reports speaker honoraria from Bayer, Novartis, Biogen, Teva, Sanofi-Aventis/Genzyme, Merck Serono, Alexion, Chugai, MedImmune, and Shire; he is advisory board member for Novartis and MedImmune Scientific and hold stocks of Nocturne GmbH; all outside the submitted work. B.W. reported grants from the Deutsche Forschungsgemeinschaft, German Ministry of Education and Research, Baden-Württemberg Ministry for Science, Research and Art, Dietmar Hopp Foundation, Klaus Tschira Foundation, grants and personal fees from Merck and Novartis, and personal fees from Alexion, INSTAND e.V., Roche; none related to this study.

Ethical standards statement All human studies have been approved by the appropriate ethics committee and have therefore been performed in accordance with the ethical standards laid down in the 1964 Declaration of Helsinki and its later amendments.

References

1. Bjornevik K, Cortese M, Healy BC, Kuhle J, Mina MJ, Leng Y, Elledge SJ, Niebuhr DW, Scher AI, Munger KL, Ascherio A (2022) Longitudinal analysis reveals high prevalence of Epstein-Barr virus associated with multiple sclerosis. Science 375:296-301
2. Chen MS, Huber AB, van der Haar ME, Frank M, Schnell L, Spillmann AA, Christ F, Schwab ME (2000) Nogo-A is a myelin-associated neurite outgrowth inhibitor and an antigen for monoclonal antibody IN-1. Nature 403:434-439
3. Eckharter C, Junker N, Winter L, Fischer I, Fogli B, Kistner S, Pfaller K, Zheng B, Wiche G, Klimaschewski L, Schweigreiter R (2015) Schwann Cell Expressed Nogo-B Modulates Axonal Branching of Adult Sensory Neurons Through the Nogo-B Receptor NgBR. Front Cell Neurosci 9:454
4. Emini EA, Hughes JV, Perlow DS, Boger J (1985) Induction of hepatitis A virus-neutralizing antibody by a virus-specific synthetic peptide. J Virol 55:836-839
5. Ferdous S, Kelm S, Baker TS, Shi J, Martin ACR (2019) B-cell epitopes: Discontinuity and conformational analysis. Mol Immunol 114:643-650
6. Gnjatic S, Ritter E, Buchler MW, Giese NA, Brors B, Frei C, Murray A, Halama N, Zornig I, Chen YT, Andrews C, Ritter G, Old LJ, Odunsi K, Jager D (2010) Seromic profiling of ovarian and pancreatic cancer. Proc Natl Acad Sci U S A 107:5088-5093
7. Guo JF, Zhang L, Li K, Mei JP, Xue J, Chen J, Tang X, Shen L, Jiang H, Chen C, Guo H, Wu XL, Sun SL, Xu Q, Sun QY, Chan P, Shang HF, Wang T, Zhao GH, Liu JY, Xie XF, Jiang YQ, Liu ZH, Zhao YW, Zhu ZB, Li JD, Hu ZM, Yan XX, Fang XD, Wang GH, Zhang FY, Xia K, Liu CY, Zhu XW, Yue ZY, Li SC, Cai HB, Zhang ZH, Duan RH, Tang BS (2018) Coding mutations in NUS1 contribute to Parkinson's disease. Proc Natl Acad Sci U S A 115:11567-11572
8. International Multiple Sclerosis Genetics C (2013) Network-based multiple sclerosis pathway analysis with GWAS data from 15,000 cases and 30,000 controls. Am J Hum Genet 92:854-865
9. Jarius S, Metz I, Konig FB, Ruprecht K, Reindl M, Paul F, Bruck W, Wildemann B (2016) Screening for MOG-IgG and 27 other anti-glial and anti-neuronal autoantibodies in 'pattern II multiple sclerosis' and brain biopsy findings in a MOG-IgG-positive case. Mult Scler 22:1541-1549
10. Jarius S, Paul F, Aktas O, Asgari N, Dale RC, de Seze J, Franciotta D, Fujihara K, Jacob A, Kim HJ, Kleiter I, Kumpfel T, Levy M, Palace J, Ruprecht K, Saiz A, Trebst C, Weinshenker BG, Wildemann B (2018) MOG encephalomyelitis: international recommendations on diagnosis and antibody testing. J Neuroinflammation 15:134
11. Jarius S, Paul F, Weinshenker BG, Levy M, Kim HJ, Wildemann B (2020) Neuromyelitis optica. Nat Rev Dis Primers 6:85
12. Jarius S, Wandinger KP, Horn S, Heuer H, Wildemann B (2010) A new Purkinje cell antibody (anti-Ca) associated with subacute cerebellar ataxia: immunological characterization. J Neuroinflammation 7:21
13. Jespersen MC, Peters B, Nielsen M, Marcatili P (2017) BepiPred-2.0: improving sequence-based B-cell epitope prediction using conformational epitopes. Nucleic Acids Res 45:W24-W29
14. Ji Z, Song R, Regev A, Struhl K (2015) Many lncRNAs, 5'UTRs, and pseudogenes are translated and some are likely to express functional proteins. Elife 4:e08890
15. Lucchinetti CF, Bruck W, Lassmann H (2004) Evidence for pathogenic heterogeneity in multiple sclerosis. Ann Neurol 56:308
16. Marrodan M, Alessandro L, Farez MF, Correale J (2019) The role of infections in multiple sclerosis. Mult Scler 25:891-901
17. Miske R, Scharf M, Borowski K, Rieckhoff N, Teegen B, Denno Y, Probst C, Guthke K, Didrihsone I, Wildemann B, Ruprecht K, Komorowski L, Jarius S (2023) Septin-3 autoimmunity in patients with paraneoplastic cerebellar ataxia. J Neuroinflammation 20:88
18. Pache F, Otto C, Wilken D, Lietzow T, Steinhagen K, Grage-Griebenow E, Schindler P, Niederschweiberer M, Wildemann B, Jarius S, Ruprecht K (2025) Broad Analysis of Serum and Intrathecal Antimicrobial Antibodies in Multiple Sclerosis Underscores Unique Role of Epstein-Barr Virus. Neurol Neuroimmunol Neuroinflamm 12:e200332
19. Reindl M, Khantane S, Ehling R, Schanda K, Lutterotti A, Brinkhoff C, Oertle T, Schwab ME, Deisenhammer F, Berger T, Bandtlow CE (2003) Serum and cerebrospinal fluid antibodies to Nogo-A in patients with multiple sclerosis and acute neurological disorders. J Neuroimmunol 145:139-147
20. Rodomonti M, Pache F, Otto C, Schindler P, Eberspacher B, Geran R, Puthenparampil M, Gallo P, Wildemann B, Jarius S, Erdur H, Ruprecht K (2025) Retrospective In Silico Analysis of Routine Laboratory Data Supports a Specific Association of Epstein-Barr Virus and Multiple Sclerosis. Eur J Neurol 32:e70430
21. Ruprecht K (2020) The role of Epstein-Barr virus in the etiology of multiple sclerosis: a current review. Expert Rev Clin Immunol 16:1143-1157
22. Safarinejad MR (2008) Evaluation of endocrine profile, hypothalamic-pituitary-testis axis and semen quality in multiple sclerosis. J Neuroendocrinol 20:1368-1375
23. Schneider-Hohendorf T, Wunsch C, Falk S, Raposo C, Rubelt F, Mirebrahim H, Asgharian H, Schlecht U, Mattox D, Zhou W, Dawin E, Pawlitzki M, Lauks S, Jarius S, Wildemann B, Havla J, Kumpfel T, Schrot MC, Ringelstein M, Kraemer M, Schwake C, Schmitter T, Ayzenberg I, Fischer K, Meuth SG, Aktas O,

Hummert MW, Kretschmer JR, Trebst C, Kleffner I, Massey J, Muraro PA, Chen-Harris H, Gross CC, Klotz L, Wiendl H, Schwab N (2025) Broader anti-EBV TCR repertoire in multiple sclerosis: disease specificity and treatment modulation. Brain 148:933-940
24. Shi M, Méar L, Karlsson M, Álvez MB, Digre A, Schutten R, Katona B, Vuu J, Lindström E, Hikmet F, Jin H, Yuan M, Li X, Yang H, Song X, Sjöstedt E, Edfors F, Oksvold P, von Feilitzen K, Zwahlen M, Forsberg M, Johansson F, Mulder J, Hökfelt T, Luo Y, Butler L, Zhong W, Mardinoglu A, Sivertsson Å, Ponten F, Fagerberg L, Lindskog C, Uhlén M, Zhang C. A resource for whole-body gene expression map of human tissues based on integration of single cell and bulk transcriptomics. Genome Biology. 2025;26(1):152.
25. Stork L, Ellenberger D, Ruprecht K, Reindl M, Beissbarth T, Friede T, Kumpfel T, Gerdes LA, Gloth M, Liman T, Paul F, Bruck W, Metz I (2020) Antibody signatures in patients with histopathologically defined multiple sclerosis patterns. Acta Neuropathol 139:547-564
26. Brooker SM, Novelli M, Coukos R, Prakash N, Kamel WA, Amengual-Gual M, Anheim M, Barcia G, Bardakjian T, Baur F, Berweck S, Bölsterli BK, Brugger M, Cassini T, Chatron N, Corner B, Dafsari HS, de Sainte Agathe JM, Ellis CA, Ezell KM, Foucard C, Frucht SJ, Garcia MC, Gill D, Guimier A, Hamid R, Heine-Suñer D, Herkenrath P, Hully M, Isaias IU, Januel L, Laurencin C, Laut T, Lavillaureix A, Lesca G, Lesieur-Sebellin M, Magistrelli L, Marelli C, Mefford HC, Mendelsohn BA, Mercimek-Andrews S, Miller C, Mohammad SS, Morgante F, Nandipati S, Opladen T, Padmanaban M, Pauni M, Pezzoli G, Piton A, Ramond F, Riboldi GM, Rougeot-Jung C, Santos-Simarro F, Scheffer IE, Serari N, Stahl CM, Kung AS, Tarongí Sanchez S, Thauvin-Robinet C, Till M, Tranchant C, Troedson C, Tropea TF, Vanakker O, Vega P, Wiese ML, Wieshmann U, Williams LJ, Wirth T, Zech M, Zempel H, Roze E, Leuzzi V, Galosi S, Fung VSC, Carvill G, Krainc D, Gerard E, Mencacci NE. The spectrum of neurologic phenotypes associated with NUS1 pathogenic variants: a comprehensive case series. Ann Neurol. 2025 Sep;98(3):561-572. doi: 10.1002/ana.27272.
27. Szklarczyk D, Kirsch R, Koutrouli M, Nastou K, Mehryary F, Hachilif R, Gable AL, Fang T, Doncheva NT, Pyysalo S, Bork P, Jensen LJ, von Mering C (2023) The STRING database in 2023: protein-protein association networks and functional enrichment analyses for any sequenced genome of interest. Nucleic Acids Res 51:D638-D646
28. Uhlen M, Oksvold P, Fagerberg L, Lundberg E, Jonasson K, Forsberg M, Zwahlen M, Kampf C, Wester K, Hober S, Wernerus H, Bjorling L, Ponten F (2010) Towards a knowledge-based Human Protein Atlas. Nat Biotechnol 28:1248-1250
29. UniProt C (2021) UniProt: the universal protein knowledgebase in 2021. Nucleic Acids Res 49:D480-D489
30. Zamecnik CR, Sowa GM, Abdelhak A, Dandekar R, Bair RD, Wade KJ, Bartley CM, Kizer K, Augusto DG, Tubati A, Gomez R, Fouassier C, Gerungan C, Caspar CM, Alexander J, Wapniarski AE, Loudermilk RP, Eggers EL, Zorn KC, Ananth K, Jabassini N, Mann SA, Ragan NR, Santaniello A, Henry RG, Baranzini SE, Zamvil SS, Sabatino JJ, Jr., Bove RM, Guo CY, Gelfand JM, Cuneo R, von Budingen HC, Oksenberg JR, Cree BAC, Hollenbach JA, Green AJ, Hauser SL, Wallin MT, DeRisi JL, Wilson MR (2024) An autoantibody signature predictive for multiple sclerosis. Nat Med 30:1300-1308
31. Zhang JH, Chung TD, Oldenburg KR (2000) Confirmation of primary active substances from high throughput screening of chemical and biological populations: a statistical approach and practical considerations. J Comb Chem 2:258-265

Authors and Affiliations

S. Jarius[1] · K. Ruprecht[1] · I. Metz[2] · F.B. König[2] · J Haas[1] · W. Brück[2] · F. Paul[3,4] · B. Wildemann[1]

✉ S. Jarius
sven.jarius@med.uni-heidelberg.de

K. Ruprecht
klemens.ruprecht@charite.de

I. Metz
imetz@gwdg.de

F.B. König
fatimabarbara.koenig@med.uni-goettingen.de

J. Haas
juergen.haas@med.uni-heidelberg.de

W. Brück
wbrueck@med.uni-goettingen.de

F. Paul
friedemann.paul@charite.de

✉ B. Wildemann
brigitte.wildemann@med.uni-heidelberg.de

1 Division of Neuroimmunology, Department of Neurology, University of Heidelberg, Heidelberg, Germany
2 Department of Neuropathology, University of Göttingen, Göttingen, Germany
3 Experimental and Clinical Research Center, a cooperation between the Max Delbrück Center for Molecular Medicine in the Helmholtz Association and Charité – Universitätsmedizin Berlin, Berlin, Germany
4 NeuroCure Clinical Research Center, Charité – Universitätsmedizin Berlin, corporate member of Freie Universität Berlin and Humboldt Universität zu Berlin, and Berlin Institute of Health, Berlin, Germany

Journal of Neurology (2026)
DOI 10.1007/s00415-026-13661-z

ELECTRONIC SUPPLEMENTARY MATERIAL

Supplementary Table 1 List of all fourteen human full-length protein constructs represented on the microarray that bear the P-(SA)-x-(SGA)-R-(SN)-(LRKH) motif

Gene name	Protein name (synonyms)	Accession code	P-(SA)-x-(SGA)-R-(SN)-(LRKH) sequence
RBMY2FP[#]	RNA Binding Motif Protein Y-Linked Family 2 Member F, hypothetical protein MGC26641	NM_144971.1	PASSRSR
CHERP*	Calcium homeostasis endoplasmic reticulum protein	NM_006387.4	PSRSRSR
SRSF1*	Splicing factor, arginine/serine-rich 1 (splicing factor 2, alternate splicing factor), transcript variant 1	NM_006924.3	PSYGRSR
SRSF7*	Splicing factor, arginine/serine-rich 7, Splicing factor 9G8	BC000997.2	PSRSRSR
SRSF8 (SFRS2B)[#]	Splicing factor, serine/arginine-rich 8, Pre-mRNA-splicing factor SRP46, Splicing factor SRp46, Splicing factor, arginine/serine-rich 2B	BC057783.1	PSCSRSR
TRA2B (SFRS10)* (not to be mixed up with SRSF10 = SFRS13A, FUSIP1)	Transformer-2 protein homolog beta, TRA-2 beta, TRA2-beta, hTRA2-beta, Splicing factor, arginine/serine-rich 10	NM_004593.1	PARSRSK
ZRANB2*	Zinc finger Ran-binding domain-containing protein 2, transcript variant 1, Zinc finger protein 265, Zinc finger, splicing	NM_203350.1	PSSSRSR
CHMP2B[#]	Charged multivesicular body protein 2b, Chromatin-modifying protein 2b, CHMP2.5, CHMP2b, Vacuolar protein sorting-associated protein 2-2, Vps2-2, hVps2-2	BC001553.1	PSAARSL
RTN2[#]	Reticulon-2, Neuroendocrine-specific protein-like 1, NSP-like protein 1, Neuroendocrine-specific protein-like I, NSP-like protein I	BC014244.1	PSPSRSR

MINK1#	Misshapen-like kinase 1, transcript variant 4, 1, 2.7.11.1, GCK family kinase MiNK, MAPK/ERK kinase kinase kinase 6, MEK kinase kinase 6, MEKKK 6, Misshapen/NIK-related kinase, Mitogen-activated protein kinase kinase kinase kinase 6	NP_056531	PAPARSL
NUS1#	Dehydrodolichyl diphosphate synthase complex subunit NUS1, 2.5.1.87, Cis-prenyltransferase subunit NgBR, Nogo-B receptor, NgBR, Nuclear undecaprenyl pyrophosphate synthase 1 homolog	NM_138459.2	PAVGRNR
CD300LF (CLM1)#	CD300 molecule-like family member F, CMRF35-like molecule 1, CLM-1, CD300 antigen-like family member F, Immune receptor expressed on myeloid cells 1, IREM-1, Immunoglobulin superfamily member 13, IgSF13, NK inhibitory receptor, CD300f	BC028199.1	PAAGRNL
FAM134C (RETR3)#	Family with sequence similarity 134, member C, Reticulophagy regulator 3	NM_178126.2	PASSRSH
EXO1*	Exonuclease 1, transcript variant 2, Exonuclease 1, mExo1, Exonuclease I	NM_130398.1	PAHSRSH

* Corresponding to peptides identified by Zamecnik *et al.* as being immunoreactive. # Proteins newly identified for this study by screening the sequences of all proteins present on the microarray for the P-(SA)-x-(SGA)-R-(SN)-(LRKH) motif. All gene and protein names follow UniProt standard nomenclature.

ELECTRONIC SUPPLEMENTARY MATERIAL

Supplementary Table 2 Histological, clinical, and serological findings in 19 patients with multiple sclerosis

Patient	Histopathological pattern	Sex	Disease activity	Treatment	Significant IgG responses at a Z factor cut-off of 0.8
#1	Pattern I	F	Rem	Betaferon	None
#2	Pattern I	F	Rem	Copaxone	None
#3	Pattern I	M	Rem	None	RBMY2FP, CHERP, SRSF1, TRA2B, NUS1
#4	Pattern I	M	Rem	None	None
#5	Pattern I	F	Rem	None	None
#6	Pattern II	F	Rem	Betaferon	RBMY2FP, CHMP2B
#7	Pattern II	F	Rem	Betaferon	RBMY2FP, CHMP2B
#8	Pattern II	F	Rem	Rebif 44	None
#9	Pattern II	M	Rem	Rebif 44	None
#10	Pattern II	F	Rem	None	SRSF8 (SFRS2B)
#11	Pattern II	F	Rem	None	None
#12	Pattern II	M	Rem	None	SRSF1, SRSF8 (SFRS2B)
#13	Pattern III	F	Rem	Mitoxantrone	RBMY2FP
#14	Pattern III	F	Rem	None	RBMY2FP
#15	Pattern III	M	Rem	None	CHMP2B, RTN2
#16	Pattern III	F	Rem	None	RBMY2FP, SRSF1
#17	Pattern III	F	Rem	None	None
#18	Pattern II	M	Rel	None	CHERP, SRSF1, CHMP2B, SRSF8 (SFRS2B), RTN2
#19	Pattern III	F	Rel	None	RBMY2FP
#18 CSF	Pattern II	M	Rel	None	RBMY2FP
#19 CSF	Pattern III	F	Rel	None	RBMY2FP

Abbreviations: F = female; M = male; Rel = relapse; Rem = remission.

Journal of Neurology (2026)
DOI 10.1007/s00415-026-13661-z

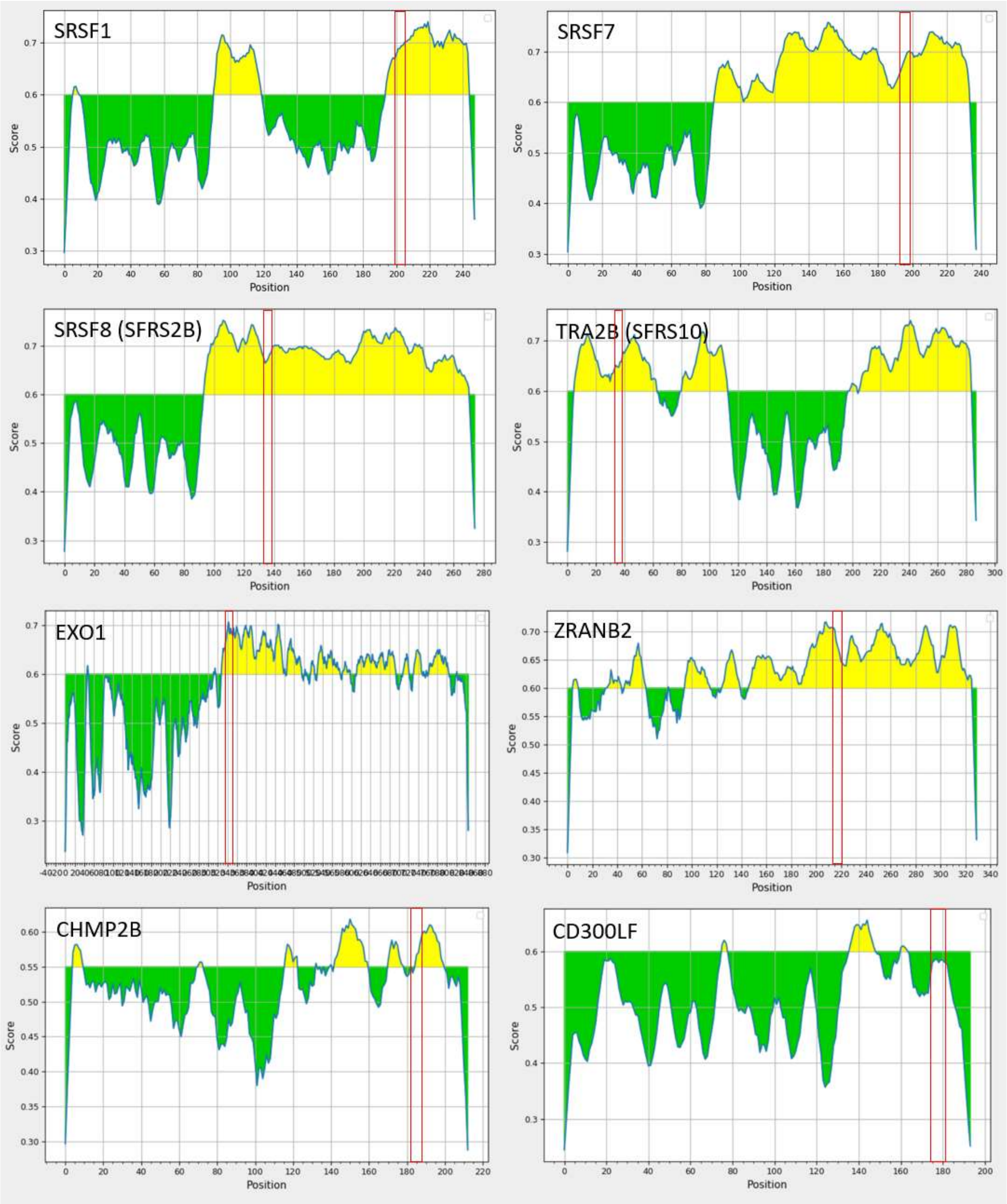

Supplementary Fig. 1 (continues on next page)

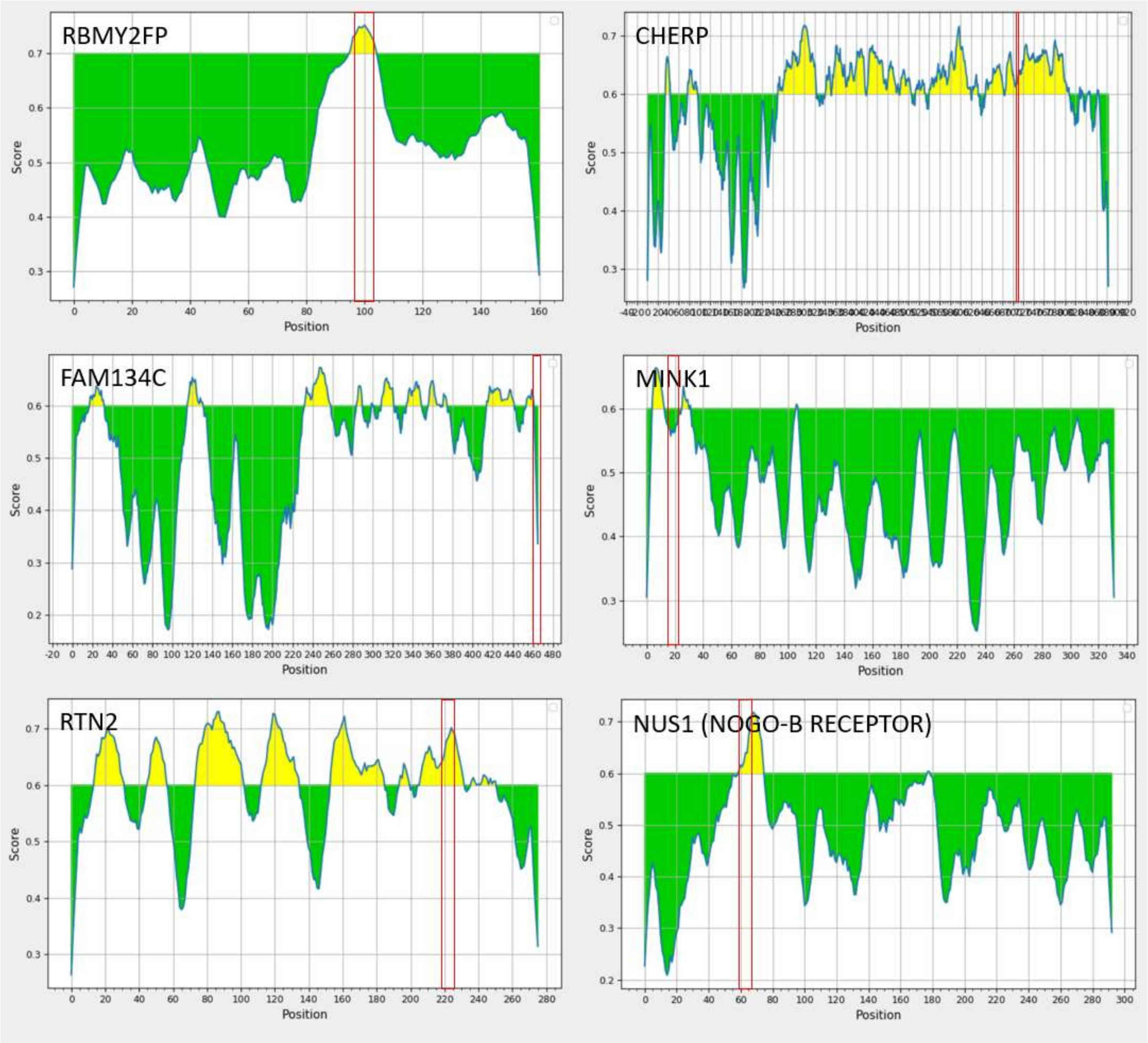

Supplementary Fig. 1 (continued) BepiPred-2.0 sequential B-cell epitope prediction analysis. BepiPred-2.0 predicts B-cell epitopes from protein sequences, using a Random Forest algorithm trained on epitope and non-epitope amino acids derived from crystal structures. Sequential prediction smoothing is applied afterwards [13]. Residues with scores above the selected threshold are predicted to be part of an epitope and are shown in yellow. The Y-axis depicts residue scores and the X-axis residue positions. A conservative threshold of 0.6 (default threshold 0.5) was applied, corresponding to a sensitivity of 0.09559 and a specificity of 0.95116 [13]. Red frames indicate the position of the P-(SA)-x-(SGA)-R-(SN)-(LRKH) motif sequence within the respective protein sequence (protein sequences as provided by the microarray manufacturer). Of particular note, in RBMY2FP and NUS1, the motif sequence exactly matches the only predicted epitope. Moreover, in all proteins that yielded a significant IgG response, the motif was located within a region predicted to be part of a B-cell epitope. Among the motif-bearing proteins that did not generate a significant IgG response in any patient in the microarray experiment at a Z factor cut-off of 0.8 (CD300LF, FAM134C, MINK1, SRSF7, and ZRANB2), the motif was located outside predicted epitope regions in CD300LF, FAM134C, and MINK1. Using a slightly less stringent cut-off of 0.7, also SRSF7 yielded three hits in the MS group and ZRANB2 at least one. In contrast, CD300LF, FAM134C and MINK1 remained negative even at a very low cut-off of 0.4 in both MS patients and controls, consistent with the motif in these proteins being located outside predicted B-cell epitopes.

Journal of Neurology (2026)
DOI 10.1007/s00415-026-13661-z

ELECTRONIC SUPPLEMENTARY MATERIAL

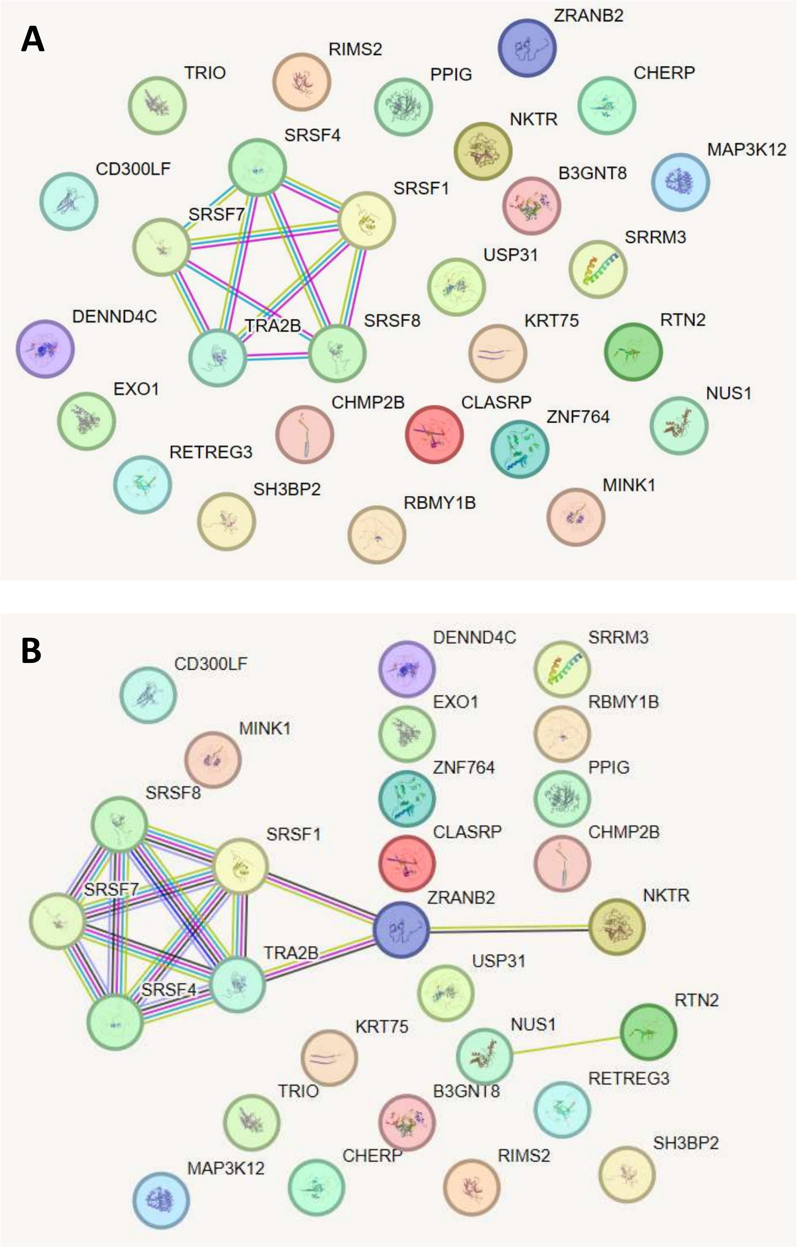

Supplementary Fig. 2 Protein-protein interaction (PPI) network of proteins bearing the P-(SA)-x-(SGA)-R-(SN)-(LRKH) motif identified in this study or previously by Zamecnik *et al.* [30], generated using STRING. A: Physical interaction subnetwork. B: Combined physical interactions and functional associations (minimum required interaction score ≥ 0.4; PPI enrichment p = 1.42×10^{-5}; 14 observed edges against 3 expected edges, indicating greater interconnectivity than expected for a random protein set matched for size and degree distribution drawn from the genome [27]). C5orf60 and LOC100652901 were not recognized by STRING and were excluded from the analysis. The displayed associations are supported by experimental evidence, curated databases, and text mining (see ref. [27] for details). In an extended STRING analysis, PPIG was additionally connected to PNMA2 (Ma2), a well-known autoantigen in paraneoplastic neurological syndromes (not shown).

DOI 10.1007/s00415-026-13661-z

ELECTRONIC SUPPLEMENTARY MATERIAL

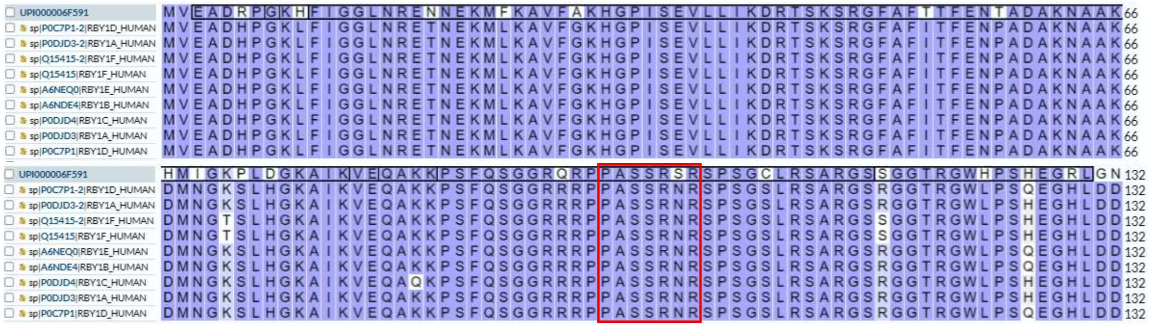

Supplementary Fig. 3 Sequence alignment of RBMY2FP and RBMY1 family members. Multiple sequence alignment demonstrates a high degree of sequence homology between RBMY2FP (row 1) and RBMY1 family proteins (rows 2–9) within the N-terminal region encompassing amino acids 1–132. Sequence alignment generated using the UniProt alignment algorithm [29]. The red frame indicates the positions of the P-(SA)-x-(SGA)-R-(SN)-(LRKH) motif.

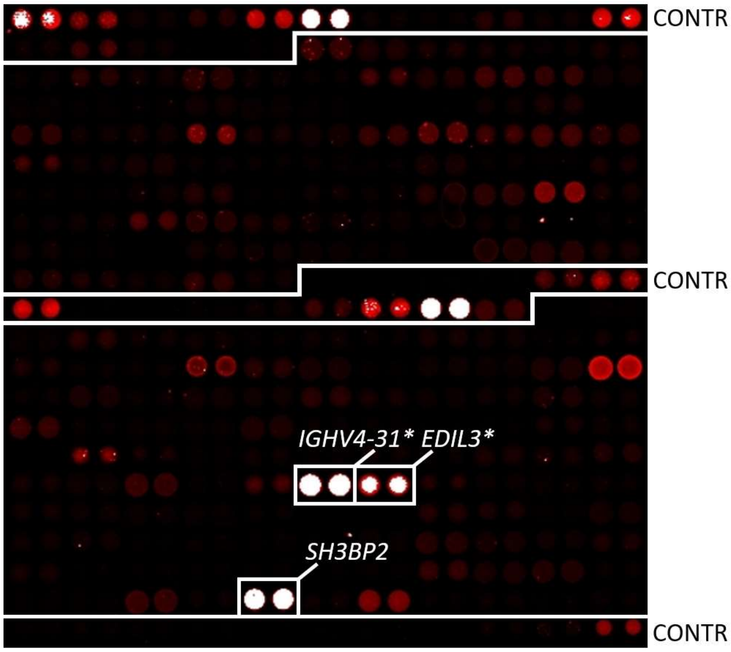

Supplementary Fig. 4 IgG response to SH3BP2 isoform 1 in a patient with MS. Strong binding of serum IgG from a patient with MS to full-length human SH3BP2 (SH3 domain-binding protein 2) was detected using the microarray. This protein corresponds to peptide 216602, which generated an IgG response in almost 40% of MS patients in the study by Zamecnik *et al.* [30]. However, the isoform used on the microarray (isoform a) does not contain the P-(SA)-x-(SGA)-R-(SN)-(LRKH) motif (present only in isoform b, corresponding to transcript variant 3). These results suggest that the immune response to SH3BP2 may be broader than expected in some patients, possibly due to epitope spreading. The image shows a magnified cut-out of the total array (block 24 of 48 blocks). Internal positive and negative control spots are marked "CONTR". Two additional antibody responses (IGHV4-31, immunoglobulin heavy variable 4-31; EDIL3, EGF-like repeats and discoidin I-like domains 3) were considered non-specific, as they are frequently observed in patients with various diseases and in healthy controls according to the manufacturer; in fact, both were detected as 'hits' in all MS and control serum samples in the cohort analysed here.
